# Soil fertility analysis in the Republic of Bashkortostan

**DOI:** 10.1038/s41598-022-26031-2

**Published:** 2022-12-20

**Authors:** Ramil Mirsayapov, Ilgiz Asylbaev, Anna Kiseleva, Tatiana Minkina, Nadezhda Kurmasheva

**Affiliations:** 1 Department of Soil Science, Agrochemistry and Precision Agriculture, Federal State Budgetary Educational Establishment of Higher Education, “Bashkir State Agrarian University”, Ufa, Russia; 2 Department of Environmental Management, Construction and Hydraulics, Federal State Budgetary Educational Establishment of Higher Education, “Ufa State Petroleum Technological University”, Ufa, Russia; 3 Scientific and Educational Center, Federal State Budgetary Educational Establishment of Higher Education, “Bashkir State Agrarian University”, Ufa, Russia; 4 Department of Soil Science and Land Resource Assessment, Federal State Autonomous Educational Institution of Higher Education, “South Federal University”, Rostov-On-Don, Russia

**Keywords:** Geochemistry, Agroecology

## Abstract

The manuscript presents the materials of soil fertility analysis of agricultural lands in the north-eastern forest-steppe of the Republic of Bashkortostan in the conditions of the Salavatskiy district of the republic (Russian Federation). Agrochemical analysis of the humus accumulation, mobile phosphorus, exchangeable potassium was carried out, morphological properties were studied, the thickness of the humus horizon, granulometric composition and soil erosion were determined. During the 49-year period of agricultural land use, it was revealed some of the medium-humus soils passed into the category of high-humus, caused by the fact that the arable lands were not used for cultivating crops and they were withdrawn from circulation and sown with many-year-old grasses. As to the thickness of the humus horizon, low-yielding soils predominate, which occupy 60.11%, average 32.9%. The main land area belongs to slightly washed soils of 67,445.2 ha (58.2%) and unwashed 36,985.5 ha (31.9%). Agricultural lands are mainly characterized as medium-humus (80.3%) and obese (12.1%) ones. Based on the results of the research, an adjustment, digitization and a new soil map of the Salavatskiy district was made with the allocation of the main types and subtypes of soils with the indication of varieties on a scale of 1:25,000.

## Introduction

World and domestic experience show that high and sustainable agricultural productivity is possible only with a comprehensive analysis of all agrochemical and environmental factors necessary for normal plant growth and development, crop formation and its quality, prevention of land degradation (acidification, salinization, overexploitation, erosion, deflation, depletion of organic matter reserves, etc.). nutrients available to plants, pollution by harmful substances, etc.). The bioclimatic potential of the agricultural lands territory in Russia is 3 times lower than in the countries of Western Europe and the USA. Therefore, for the conditions of our country, it is especially important to ensure agroecological conditions favourable for plants to carry out appropriate agrotechnical, agrochemical, reclamation and other measures aimed at improving not only agrochemical but also physical, water-physical and biological properties of soils of agricultural lands based on the results of comprehensive monitoring of soil fertility. The soil properties are in interaction with each other. An integrated approach to the assessment of soil fertility taking into account the values of integral indicators of all the main soil properties that determine plant productivity, makes it possible, at the lowest cost, based on the established limiting factors, to increase the soil fertility of each specific land plot. Soil fertility is the basis for the creation of a large volume of biomass which determines many useful functions of the soil in urbanized areas^1,2^.

Taking into account the fact one of the main requirements of efficient production is the formation of working areas from facies close in potential and effective fertility, especially in agricultural lands with a negative energy balance, the differentiation of agrochemical indicators of the arable horizon can be corrected by the introduction of precision farming technology or its elements^3,4^.

The Federal Law of the Russian Federation “About State Regulation of ensuring the fertility of agricultural lands” defines the conduct of soil, agrochemical, phytosanitary and ecological-toxicological surveys and monitoring of soil fertility of agricultural lands as one of the main directions of agrochemical services. In the field of ensuring soil fertility this law defines as the most important scientific research on the development of indicators of the fertility state of agricultural land, taking into account the natural and agricultural zoning of land, as well as methods for assessing the state of land and taking into account indicators of their fertility state^5^.

The importance of preserving soil fertility has been widely recognized over the past 200 years^6^. A separate Commission on Soil Fertility and Plant Nutrition was established in the International Society of Soil Scientists^7^. The US Forest Service defines soil fertility as its productivity, the ability to ensure plant growth due to the combination of physical, biological and chemical properties of the soil, including the content of organic matter, nutrition elements, acidity, granulometric composition, profile power and water retention capacity^8,9^.

The productivity of agricultural crops, obtaining high and stable yields depends greatly on soil fertility, where all the energy of the upper layer of the Earth is accumulated. Thus, the preservation and reproduction of soil fertility becomes the most important task in ensuring food and environmental security of the country. During agricultural activity, organic and mineral compounds are removed from the soil in large quantities which leads to a decrease in fertility, and in conditions of intensive agriculture it requires additional costs to maintain it at an optimal level^10^. The identification of anthropogenic changes in the soil is provided by soil monitoring, the main task of which is systematic observations of changes in agrochemical, agrophysical and other soil indicators, including the use of GIS technologies with the results of remote sensing of the Earth^11–14^. Monitoring the state of the soil cover makes it possible to identify degradation processes, develop measures to prevent negative processes and increase the efficiency of agricultural production^15^. However, monitoring the state of the land using remote sensing with its wide access does not make it possible to give an objective picture of soil fertility, but it only provides a superficial assessment. That is why, for a deeper study of soil properties indicators, it is recommended to conduct forwarding and field soil surveys, laboratory agrochemical analysis and import data into geographical information systems to solve emerging problems^11,16–18^. Such surveys provide systematization of soil fertility indicators, their moving into a single electronic database which is necessary for land use management and planning^19^. Accounting and assessment of the soils state is mandatory for the placement of field crops, the calculation of tax payments on land, taking into account soil fertility and, of course, for the correct and rational use of agricultural land. Up to date, the quality of the materials of previous studies conducted in 1970–1980 used in the assessment and mapping are ineffective in energy and production costs^20^. A large variety and volume of agrochemical data from past soil surveys are mostly inaccessible for work^21,22^.

The purpose of the research was to monitor the state of soil fertility with the compilation of the original electronic soil map of agricultural lands in the Salavatskiy district of the Republic of Bashkortostan, a retrospective analysis of the agricultural land state. The research was carried out as part of the implementation of the Program “Eurasian carbon landfill” for the creation and operation of a carbon landfill on the territory of the Republic of Bashkortostan.

The objectives of the research were to conduct a soil survey of agricultural lands, which includes the determination of the most important agrochemical indicators of soils, their morphological description. Ultimately, it is the compilation of an up–to-date electronic soil map of the Salavatskiy district of the Republic of Bashkortostan.

## Research objects

**Figure 1:**
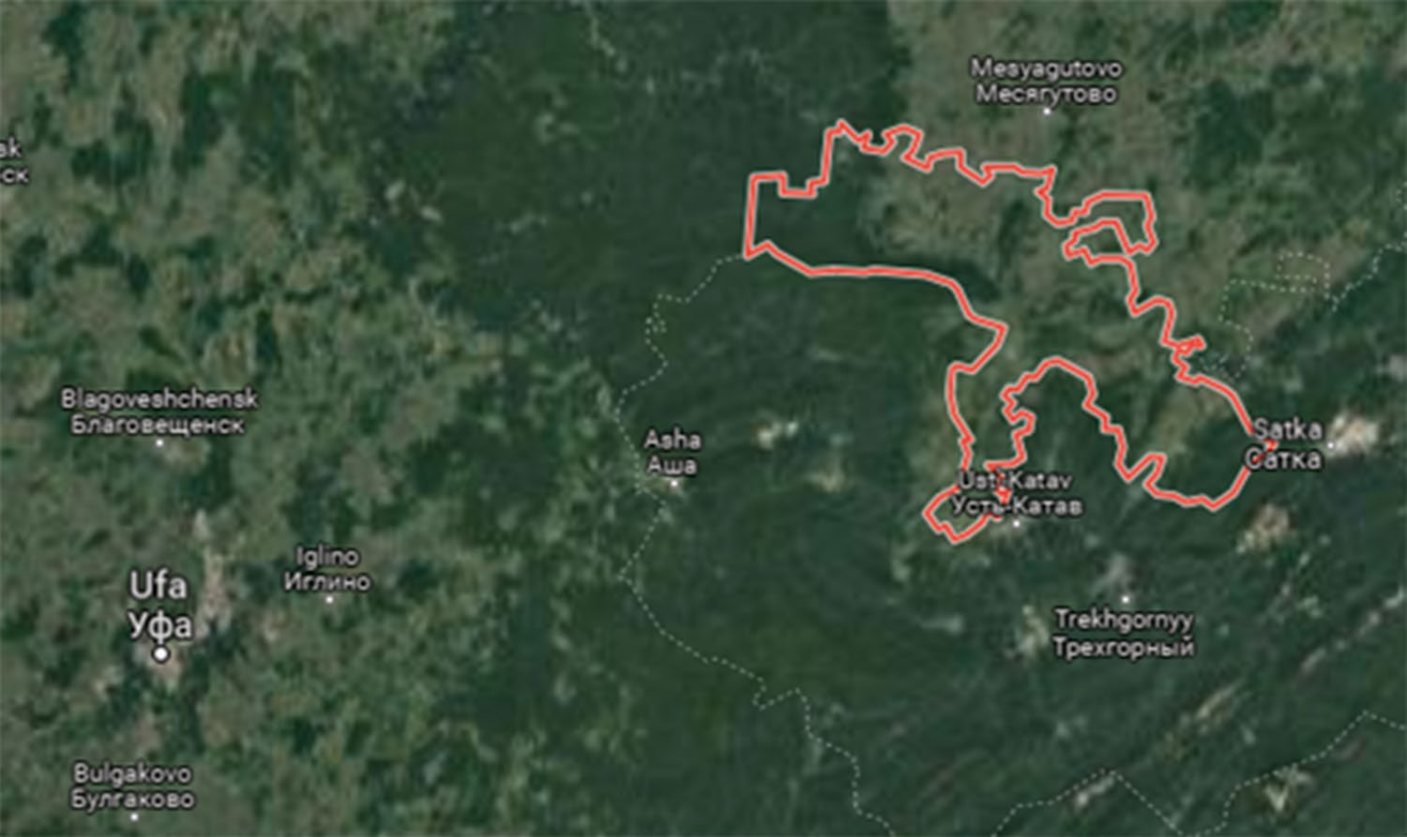
Location of the area of the studied territory.

The objects of research are the soils of agricultural lands of the Republic of Bashkortostan (Russian Federation), located 55°13′17″ north latitude 58°09′00″ east longitude, covers an area of 113,460 hectares (Fig. 1).

The territory of the Salavatskiy district of the Republic of Bashkortostan belongs to the geomorphological region of the Yuryuzano-Aiskaya foothill plain with an altitude of 200–300 m above sea level, and in the natural and agricultural zone to the north-eastern forest-steppe. The main part of the territory is located on the watershed of the Yuryuzan and Ai rivers^23^. The advanced ridges of the western slope of the Urals stretch along the south-eastern part (Suleya, Bashkir Ilchikeyevo). The rocks are composed of limestones, marls, sandstones, gypsum of the Kungur tier. The Karatau ridge rises in the north, the Ufa Plateau is located in the northwest. The average surface height is 370 m. The climate is sharply continental, moderately cool and humid. The average annual air temperature is 1.5 °C, the average temperature in January is 16 °C below zero, in July it is 17 °C. The inversion of temperatures is expressed in the values. The average annual number of sieges is 650 mm.

Due to the fact that the district is located within the forest-steppe zone, secondary birch forests were preserved, in the central part arable and settled meadows predominate in place of pine forests, in the south—southern taiga pine forests largely replaced by secondary birch and aspen species, as well as foothills forests represented by light–coniferous broad-leaved and birch cultures^23^.

## Research methods

The survey of the territory consisted of 4 stages: stage 1 (preparatory)—the materials of the 1972 survey were collected and digitized, a layout of a digitized soil map was compiled in the MapInfo program, on which an approximate network of traffic routes was marked; stage 2 (field) – soil sections were laid and marked with GPS coordinates by the Garmin Montana 680 navigator, the boundaries of eroded lands were being clarified according to the “All-Union Instruction on soil surveys and the compilation of large-scale soil maps of land use”. A topographic survey of the surveyed territory was also carried out on a scale of 1:25,000. Soil maps were compiled using MapInfo, ArcGIS 10.4.1 and Adobe Illustrator CS5. 3 stage (laboratory-analytical) – agrochemical analysis was carried out for the content of organic matter in the soils of the Tyurin method (GOST 26,213-91), mobile phosphorus and potassium compounds—by the Chirikov’s method in the modification of Pryanishnikov Institute of Agrochemistry (National State Standard 26,204-91), pH salt extraction – by the potentiometric method (National State Standard 26,483-85)^24^. Soil samples were selected according to the main genetic horizons of the soil profile after the laying of the soil section, including the humus-accumulative horizon (A), illuvial (B) and soil-like rock (C). When selecting soil samples, the laws of the formation of soil cover in landscapes were taken into account. Genetic horizons were visually isolated, soil samples weighing 300 g were taken and their laboratory analysis was carried out for the content of humus, acidity, mobile forms of phosphorus and potassium, granulometric composition after drying and sieving through sieves with a diameter of 0.25 mm. The selection of soil samples was carried out in triplicate. Determination of agrochemical and morphological properties.

The content of organic matter in soils by the Tyurin method (Russian National Standard 26,213-91). The method is based on the oxidation of organic matter with a solution of potassium bicarbonate in sulfuric acid and the subsequent determination of trivalent chromium equivalent to the content of organic matter on a photoelectrocolorimeter. A representative sample weighing 3–5 g is taken from the ground soil or rock for fine grinding. Before grinding, undecayed roots and plant residues seen by an aided eye are removed from the sample with tweezers. Then the sample is completely crushed and passed through a braided sieve with holes with a diameter of 0.25 mm. For fine grinding, mortars and grinding devices made of porcelain, steel and other hard materials are used. Then, the soil sample is analyzed.

Determination of mobile compounds of phosphorus and potassium – by the Chirikov method in the modification of the Head Scientific and Methodological Centre for the Agrochemical Service (Russian National Standard 26,204-91). This standard establishes a method for determining mobile phosphorus and potassium compounds in black soils, gray forest and other soils, overburden and host rocks of steppe and forest-steppe zones. The method is based on the extraction of mobile compounds of phosphorus and potassium from the soil with a solution of acetic acid concentration of mole/dm with a ratio of soil to a solution of 1:25 and subsequent determination of phosphorus in the form of a blue phosphorus-molybdenum complex on a photoelectrocolorimeter and potassium on a flame photometer.

Determination of the pH of the salt extract by the potentiometric method (Russian National Standard 26,483-85). This standard establishes a method for the preparation of salt extract from soils, overburden and host rocks for the determination of exchangeable acidity, exchangeable (mobile) aluminum, exchangeable calcium, exchangeable (mobile) magnesium, exchangeable ammonium and manganese, nitrate content, mobile sulfur and determination of its pH during soil, agrochemical, land reclamation surveys, control over the state of soils and other survey and research work. The inaccuracy of the method in determining the pH is 0.1 pH units. The essence of the method consists in the extraction of exchange cations, nitrates and mobile sulfur from the soil with a solution of potassium chloride concentration of 1 mol/dm (1 n.) at a ratio of soil and solution of 1:2.5 and potentiometric determination of pH using a glass electrode. When determining the pH in samples of organic soil horizons, the extract is prepared at a ratio of soil and solution of 1:25. The method is not suitable for determining other indicators in samples of organic soil horizons. Soil samples submitted for analysis are brought to an air-dry state, crushed, passed through a sieve with round holes with a diameter of 1–2 mm and stored in boxes or bags. The sample for analysis from the box is taken with a spoon or a spatula, having previously mixed the soil to the entire depth of the box. The soil is poured out of the bags onto a flat surface, thoroughly mixed, distributed with a layer of no more than 1 cm. A sample for analysis is taken from at least five places. Sample weight is 30 g.

Determination of the granulometric composition of soils by pipette method. Preparation of the soil for analysis. From the air-dry soil sifted through a sieve with holes of 1 mm, 10 g is weighed (with an accuracy of 0.01 g) and placed in a porcelain cup with a diameter of 10–12 cm. A 4% solution of sodium pyrophosphate is poured into a cup: for non—carbonate, unsalted, non—gypsum soils of light mechanical composition is taken 5 ml of solution per 10 g of soil; for heavy loamy, clay and carbonate soils—10 ml; for saline and gypsum—20 ml. If half-liter cylinders are used, then the soil weight and the amount of sodium pyrophosphate are halved. The soil in a porcelain cup is moistened with a solution of pyrophosphate to a dough—like state and gently rubbed with a pestle with a rubber tip for 10 min. The remainder of the pyrophosphate solution is poured into a cup with soil, added distilled water, stir and transferred to a liter cylinder through a sieve with 0.25 mm holes inserted into a glass funnel. Stirring with the addition of new portions of water is continued until the entire soil is transferred to the measuring cylinder. The volume of the suspension in the cylinder is adjusted to 1 L and analyzed by the pipette method. The soil on the sieve (coarse and medium sand) is washed with water from the washer and washed into a porcelain cup. From the cup, the soil is transferred by decanting with water without loss into a pre-weighed drying cup. Excess water is drained from the cup, the remainder is evaporated on an eternit tile, then dried in a drying box at 105 °C to a constant mass and the content of coarse and medium sand is calculated.

Preparation for sampling. Samples from the cylinder are taken with a special pipette from different depths and at different intervals. In total, four samples are taken (Table 1). The timing of sampling depends on the density of the solid phase of the soil and the temperature of the suspension. The density of the solid phase is determined in advance or use approximate data (g/cm3): for soils of light granulometric composition, as well as humus and arable horizons with a humus content of less than 5%—2.6; for humus and arable horizons with a humus content of more than 5% (podzolized, leached, typical and ordinary black soils)—2.4; for the remaining underlying mineral horizons with a humus content of less than 1%—2.7.

**Table 1:** Land areas by humus supply.

No	Humus content	According to the results of the soil survey of 2021		According to the results of the 1972 soil survey	
		ha	%	ha	%
1	Low—humus (low-humus)	67.1	0.06	128	0.1
2	Thin—humus	666.91	0.6	1258	1.0
3	Medium—humus	93,137.96	80.3	106,208	80.0
4	High—humus	13,916.94	12.1	14,357	10.8
5	Soils not included in the gradation	8107.35	7.0	10,800	8.1
Total		115,896.2	100.00	132,751	100

Stage 4 (cameral) – the names of soil varieties are specified according to the “Classification and diagnostics of soils of the USSR” (1977), the areas of agricultural land are calculated^25^. A topographic survey of the analized area was carried out on a scale of 1:25,000. The mapping of the analyzed territory was carried out using the software products ArcGIS 10.4.1, MapInfo and Adobe Illustrator CS5. The data obtained as a result of the survey were processed by statistical methods using Microsoft Office Excel 2003 and Statistica 10.0^25^.

## Results and discussion

Soil studies were carried out on 115,896.2 hectares of agricultural lands in fifteen villages of the municipal district obtained by subtracting from the available area of the village industrial lands, populated areas, forest plots occupied by water, etc.

As a result of the land reform and redistribution of land for various purposes for the period from 1972 to 2021, the area of agricultural land decreased by 12.7% compared to the data of the previous survey.

In the research area, the largest territories are occupied by black soils, which amount to 52,826.24 ha, including bleached soils—42,605.9 ha, alkaline – 6983.8 ha and shortened – 3236.54 ha. Slightly inferior to the black soils are dark gray forest soils with an area of 37,043.63 hectares, alluvial—12,287.4 hectares, gray forest—6371.96 hectares and forest soils of a rooted profile – 5058.94 hectares. The share of sod-carbonate soils accounts for 7792.7 hectares of land, which is 6.2%. The gradation did not include the soils of the ravine-beam complex, sand and gravel masses, existing ravines and disturbed lands, and quarries that occupy 5,452.4 hectares of territory (4.3%).

One of the important indicators of soils, especially used in agricultural production, is the humus state. Thus, over 49 years there has been a slight decrease in the area under obese (high-humus) soils in the hectare ratio, due to a general decrease in the area of farmland, but in the context of the security group, they have increased by 1.3% (Table 1). The remaining levels of security have remained almost at the same level. The increase in the amount of fat chernozems was facilitated by the withdrawal of arable land from circulation and their transfer to perennial plantations. Earlier researches conducted on experimental fields of the Bashkir State Agrarian University identified and revealed changes in the quantitative and qualitative composition of organic matter from 15 to 30% when introducing a land plot for arable land^26^. To preserve and improve soil fertility, it is recommended to carry out a complex of agrotechnical, agrochemical and reclamation measures and the use of various meliorants, organic and mineral fertilizers^27^.

**Figure 2:**
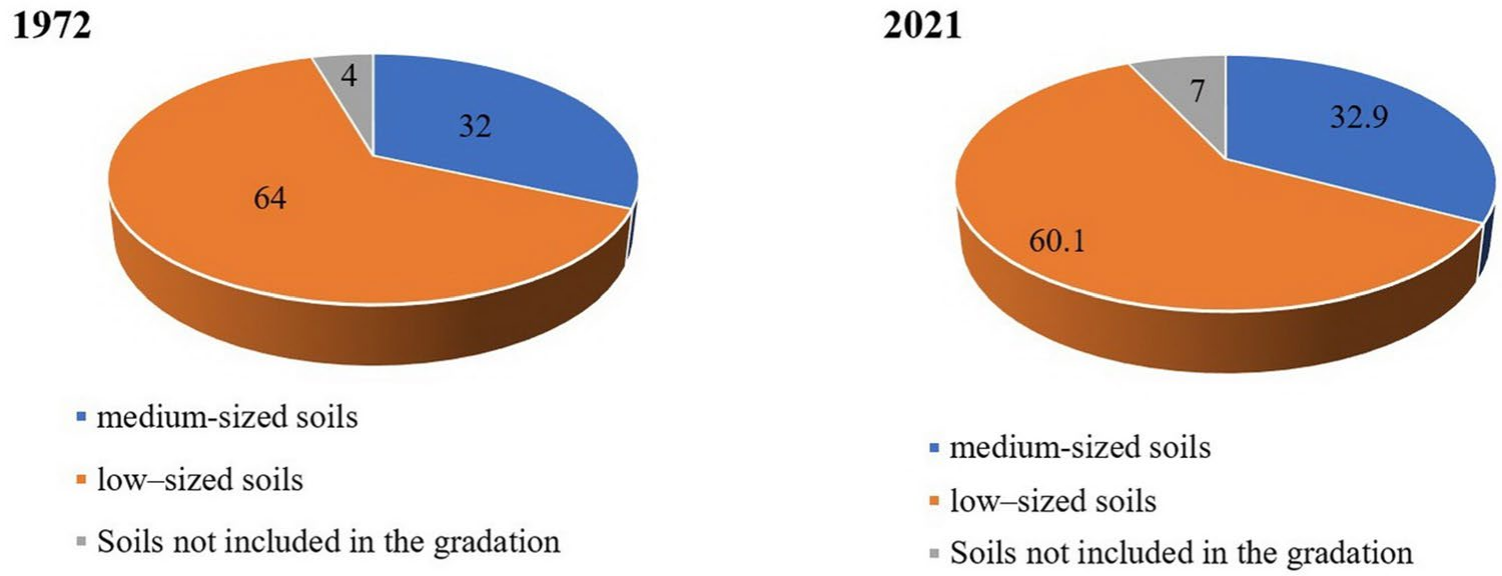
Distribution of soils by humus horizon thickness by region.

Studies of the capacity of the humus horizon have shown that low–sized soils have become the most widespread—69,660.2 hectares or 60.1% of the total area of agricultural land (Fig. 2). A smaller area is occupied by medium-sized soils – 38,128.7 hectares (32.9%), not included in the gradation – 8107.3 hectares or 7.0%, respectively. It should be noted that the specific gravity of the soil of the ravine-beam complex, sand and gravel masses, active ravines and disturbed lands, and quarries increased by 2.5%.

**Figure 3:**
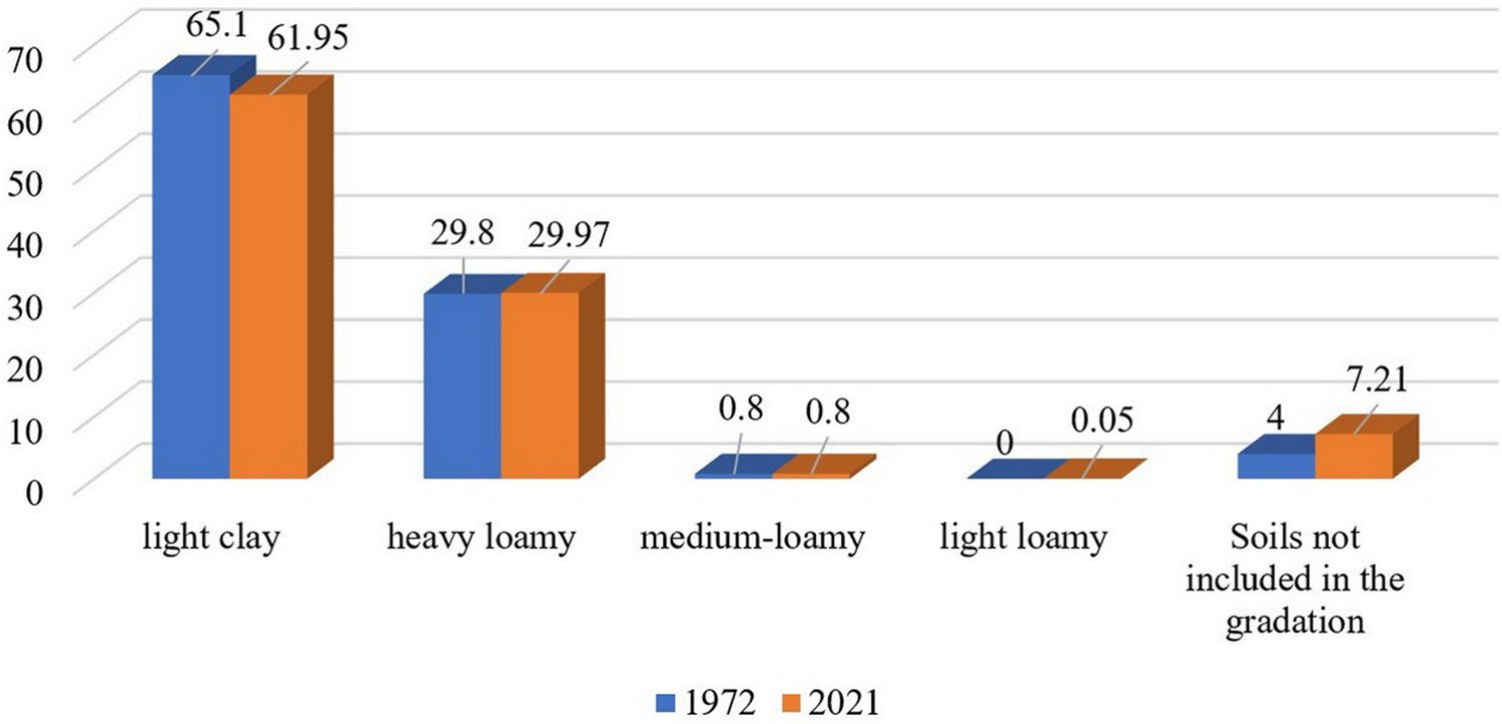
Distribution of Salavatskiy district soil areas by granulometric composition, %.

The granulometric composition of the soil is also of great agronomic importance^28^. Physical, physico-chemical, physico-mechanical properties and water, air, and nutrient regimes of soils depend on it^29,30^. In the Salavatskiy district there were practically no changes in soil areas in terms of granulometric composition, mainly clay soil varieties predominate. According to the mechanical composition of the soil there were distributed as follows: light clay – 71,807.38 ha or 62% (in 1972, 86,375 ha or 65.1%) of the total area of agricultural land and heavy loamy – 34,745.24 ha (30%) (in 1972—39,614 ha or 29.8%). The share of medium-loamy varieties accounts for 0.8% (in 1972—0.84%) (Fig. 3).

The gradation did not include 8362.27 hectares of land. Heavy loamy, medium clay, sandy loam and sandy soils have not been identified.

All arable soils of the analyzed territory are slightly susceptible to erosion processes, the processes of water and, to a lesser extent, wind erosion have developed. 67,445.21 hectares of land, or 58.2% (in 1972, 77,702 hectares) of the total area of agricultural lands are occupied under lightly washed soils, the share of medium and heavily washed accounts for 3.9% and 0.1%, respectively. Unwashed soils are distributed on 36,985.46 hectares (31.9%) (Table 2).

**Table 2:** Soil areas by category of erosion feature (Salavatskiy district of the Republic of Bashkortostan).

No	Erosion feature	According to the results of the soil survey of 2021		According to the results of the 1972 soil survey	
		ha	%	ha	%
1	Heavily washed	111.9	0.1	112	0.1
2	Medium washed	4508.9	3.9	5254	3.9
3	Lightly washed	67,445.2	58.2	77,702	58.6
4	Unwashed	36,985.5	31.9	44,488	33.5
5	Soils not included in the gradation	6844.8	5.9	5192	3.9
Total		115,896.2	100.00	132,751	100

**Figure 4:**
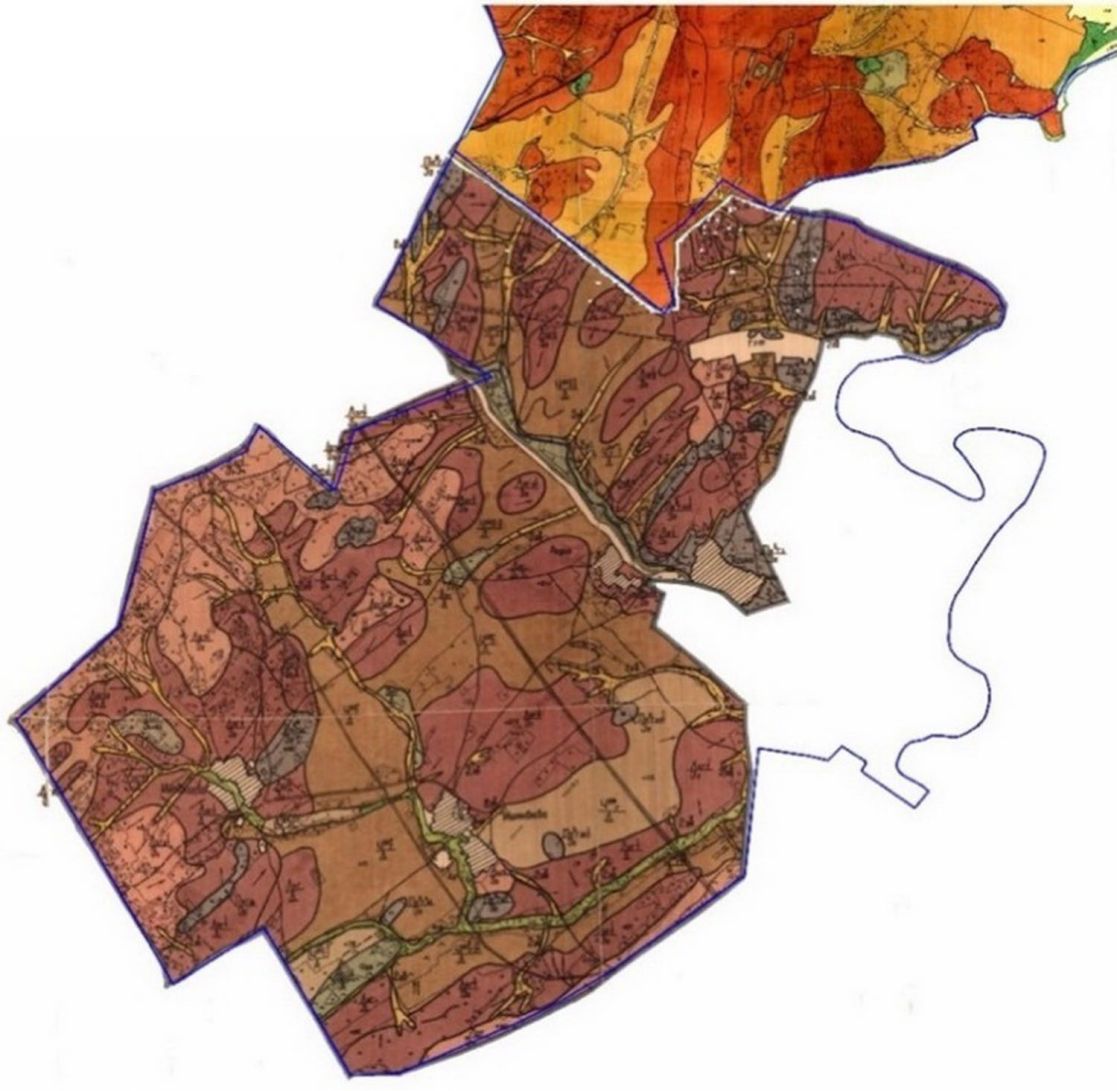
Soil map within the boundaries of the Salavatskiy district of the Republic of Bashkortostan, 1972.

According to the results of the field research and laboratory agrochemical analyses of soils, land refinements related to agricultural land were carried out. The basis for correcting and digitizing the contours of soil varieties were in the maps made in 1972 (Fig. 4).

**Figure 5:**
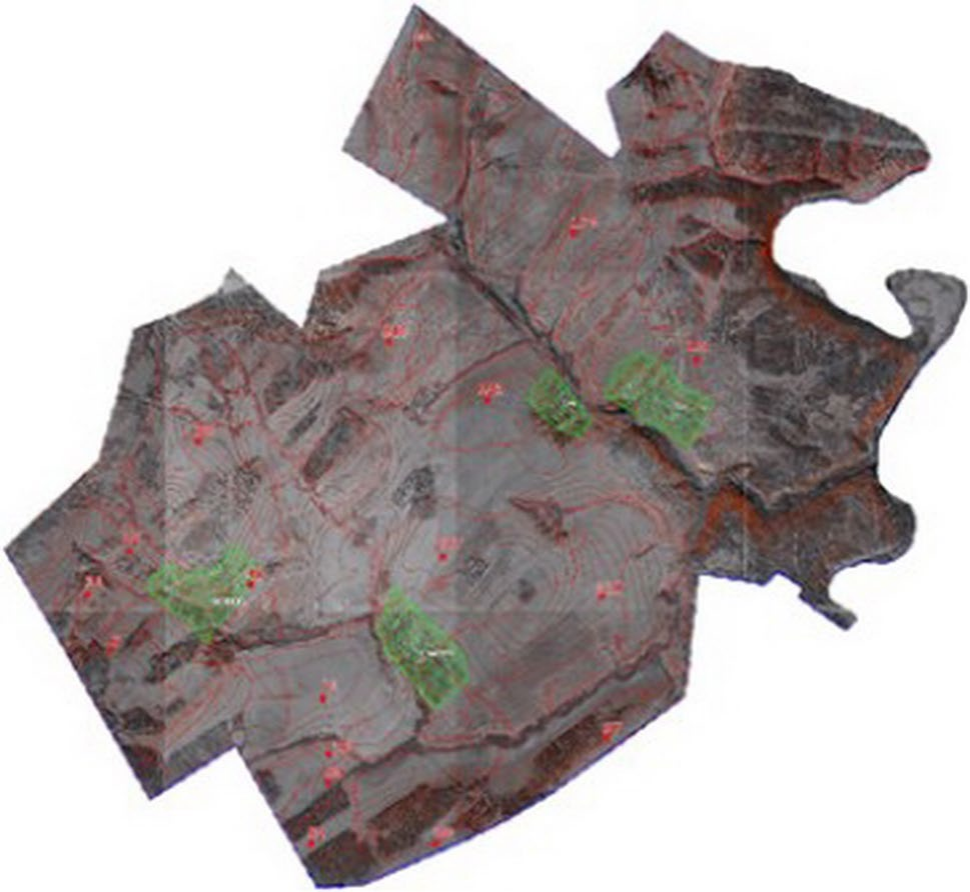
Orthophotoplan within the boundaries of the Salavatskiy district of the Republic of Bashkortostan, 2007.

Digitization included scanning the topographic basis, then assigning coordinates to a raster image, decrypting and digitizing orthophotos (Fig. 5).

After the carried-out activities, a soil map was obtained in the digital format of the Mapinfo program, after which it was converted into a raster basis with reference to the local coordinate system MSK 02 zone 1. The digitization of the 1972 soil map was carried out manually by outlining the contours of the topographic base and the scanned map.

During digitization, information partially lost due to its wear and distortion during scanning was restored. A necessary condition is the use of the originals of the soil maps of the previous survey (1972).

**Figure 6:**
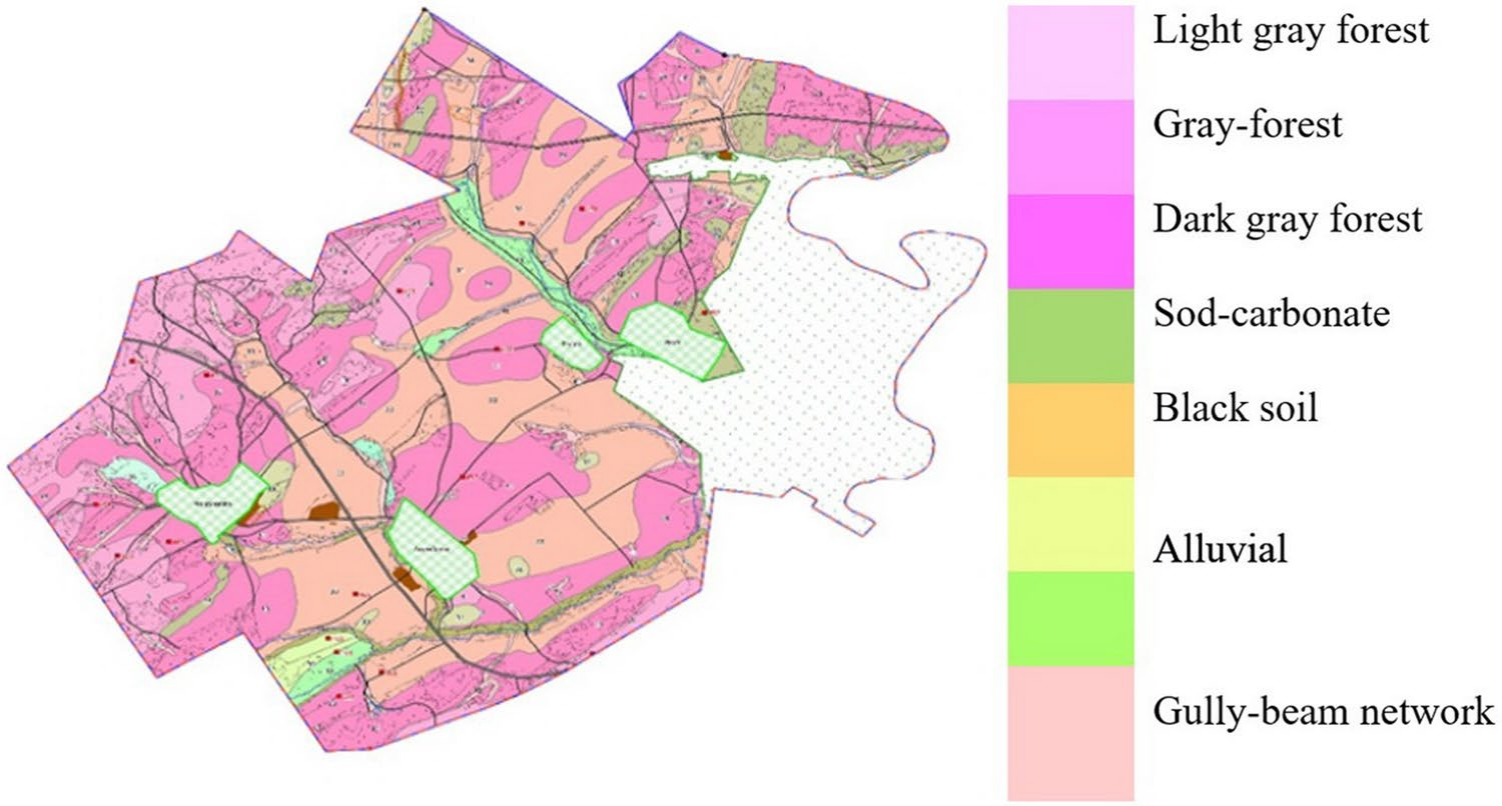
Completed soil map within the boundaries of the Salavat district of the Republic of Belarus, 2021.

As a planned basis on which the created layers can be opened and information on soils can be obtained, a raster basis was ordinated into a local coordinate system (Fig. 6).

The result of the conducted research is the developed electronic digital soil map of the municipal district of Salavatskiy district which unites 15 rural settlements. The electronic soil map is presented in the form of a complex of electronic layers with the names of the type and subtype of soils, soil variety, mechanical or granulometric composition, soil-forming and underlying rocks. It also includes indicators of organic carbon, humus, mobile phosphorus, exchangeable potassium, soil acidity by pH value and the capacity of the humus-accumulative horizon.

## Conclusion

1. The conducted comprehensive survey showed that the territory of agricultural lands includes 115,896.2 hectares, of which arable land – 55.2%, pastures – 16.8%, hayfields – 8.1%. The soil cover is represented by 14 types and 36 subtypes of soils, the largest areas are occupied by black soils—41.6% and gray forest – 38.19%.

2. The soils of the district are characterized as medium-humus and high–humus, over a 49-year period, an increase in the humus content was revealed, which is associated with the transfer of land for perennial grasses. According to the size of the humus horizon, the most widespread are low-sized soils, the least are medium-sized ones. According to the granulometric composition, light clay soils predominate. According to the degree of erosion, lightly washed and unwashed soils are common.

3. The conducted soil survey provided spatial information about the state of agricultural lands, the obtained materials served as the basis for the creation of digitization and correction of soil maps. Digitized maps, taking into account the current state of soil fertility, make it possible to develop projects for inter-household and on-farm land management of organizations of the agro-industrial complex, make a cadastral assessment of agricultural lands, determine the tax according to soil fertility and develop measures to increase their fertility.

## Data Availability

Data will be available on reasonable request from the corresponding author (Ramil Mirsayapov).
